# Betapapillomaviruses in the anal canal of HIV positive and HIV negative men who have sex with men

**DOI:** 10.1186/1471-2334-14-S4-O13

**Published:** 2014-05-29

**Authors:** Mario Poljak, Boštjan Kocjan, Boštjan Mlakar, Lea Hošnjak, Kristina Fujs Komloš, Miloš Milošević, Katja Seme

**Affiliations:** 1 Institute of Microbiology and Immunology, Faculty of Medicine, University of Ljubljana, Slovenia; 2 Department of Proctology, Surgical Centre Zdrav Splet, Maribor, Slovenia; 3 Department of Surgery, The Rožna dolina Surgical Centre, Ljubljana, Slovenia; 4 Department of General and Abdominal Surgery, General Hospital, Slovenj Gradec, Slovenia

## 

Human papillomaviruses from the genus *Betapapillomavirus* (beta-PV) are typically detected in skin and hair follicle specimens, but recently also from oral cavity, head and neck region, and esophagus. In this study, we investigated the prevalence and persistence of anal beta-PV infection and beta-PV type distribution in a national cohort of men who have sex with men (MSM) and their correlations with subject’s HIV-1 serostatus and demographic and behavioral risk factors. To the best of our knowledge, this is the first study to investigate and characterize anal beta-PV infections in MSM population as well as in general.

The study included 135 males from Slovenia with a history of receptive anal sexual intercourse. The study subjects’ ages were 17-81 years (median age=31 years) and 23/135 (17.0%) were HIV-1 positive. Detection and typing of beta-PVs was initially performed using a commercially available Diassay RHA Kit Skin (beta) HPV assay that enables simultaneous identification of 25 different beta-PV types. All negative samples or samples with undetermined beta-PV type were additionally tested with an in-house nested Ma/Ha PCR, targeting approximately 450-bp L1 gene fragment of different beta-PVs.

Beta-PV DNA was detected in 116 (64.1%) out of 181 anal samples included in the study. Infection with a single HPV type was found in 68 samples, and with multiple HPV types in 36 samples; the number of beta-PV types in multiple infections ranged from 2 to 9. Altogether, 31 distinct beta-PVs were detected, including four potentially novel HPV types. HPV-23 and HPV-38 were the most prevalent types, followed by HPV-36, HPV-24, and HPV-93; together these five types accounted for 38.3% (72/188) of all beta-PVs. Beta-PV was detected in 66/112 (58.9%) HIV-1 negative and 22/23 (95.7%) HIV-1 positive men (P<0.005). HIV-1 positive subjects had higher average number of beta-PV types (1.60 ± 0.29 vs. 0.95 ± 0.13, P<0.05). Average number of detected HPV types was higher (1.68 ± 0.36 vs. 0.75 ± 0.11, P<0.005) among MSM who used alkyl nitrates (poppers) during sexual intercourse.

This study showed that anal beta-PV infection was highly prevalent among Slovenian MSM population. HIV-1 positive status, promiscuity and use of alkyl nitrates were clearly associated with the higher prevalence of anal beta-PV infection. The clinical significance of the presence of beta-PVs in the anal canal needs to be clarified in further studies.

